# Selection of learning strategies supported on SMAA-M

**DOI:** 10.1016/j.heliyon.2022.e08978

**Published:** 2022-02-18

**Authors:** Rafael Guillermo García-Cáceres, Arnoldo Emilio Delgado-Tobón, John Wilmer Escobar-Velásquez

**Affiliations:** a School of Industrial Engineering, Universidad Pedagógica y Tecnológica de Colombia (UPTC), Sogamoso, Colombia; b School of Industrial Engineering, Universidad Militar Nueva Granada, Bogotá, Colombia; c School of Industrial Engineering, Universidad del Valle, Cali, Colombia

**Keywords:** Pedagogical strategies, Color theory, Decision making process, SMAA-M

## Abstract

The present work introduces a systematic decision making process which, based on Stochastic Multicriteria Acceptability Analysis – Matching, is aimed at supporting the selection of pedagogical strategies according to the theoretical paradigms provided by the Color Theory and the Learning Styles concept. This novel procedure is illustrated by an example which allowed comparison with the traditional decision mechanism. The results show that the innovation is valuable for case, since it allows a more tuned-to-reality solution that prioritizes relevant pedagogical strategies and discards insignificant ones. Another underlying advantage of this novel process as compared to the traditional one is the possibility it offers to develop a broader and more detailed analysis, since it provides both the set of pedagogical strategies for a course or group of students and a personalized analysis for each student, thus facilitating the teacher's work.

## 1 Introduction and literature review

In recent years, many Educational Institutions have been expressing their interest in competency-based training and active pedagogy as useful tools for their mission. With the aim of facilitating more effective classroom educational processes, the current article presents an innovative educational procedure, making use of both Herrmann's Theory of Colors about integral (whole brain) thinking [1, 2] and [3, 4, 5, 6, 7] student learning factors and resulting learning styles. These theoretical paradigms are newly supported here by Stochastic Multicriteria Acceptability Analysis – Matching (SMAA-M). Said analysis provides technical support for the selection of pedagogical strategies [8, 9, 10].

Multi-criterion methods are capable of aiding decision making (DM) processes by evaluating preference information supplied by DMs [11]. Nonetheless, this aid is sometimes limited when the DMs find it difficult to overtly express their preferences. An interesting contribution to this problem was provided by [12] and further developed by Bana e Costa [13, 14]. Through these works, the latter authors actually formalized the SMAA method, which bases the DM process on three well defined variables. On these grounds, the method has been fruitfully improved in several aspects, including its generalization to a multi-dimensional space [15, 16]. More recently, and depending on the specific nature of the matrix that plots the preference value information, new processing mechanisms have been developed, such that they are capable of managing more sophisticated data. In reviewing the evolution of SMAA [17], observed that the method has followed two main evolutionary trends, namely “pure” and “hybrid” SMAA versions. While the latter have blended SMAA with auxiliary methods, the former have revolved around the type of information handled by the procedure in question. The most recent version of the technique, known as SMAA-M [18], is actually one of the “pure” SMAA versions. As such, it is the first MCDM technique requiring the aid of a conceptual model, which is employed as theoretical paradigm to modulate the interpretation of the results.

SMAA-M is based on the contrast between its mathematical structure and a theoretical framework that varies depending on the case. This allows the technique to determine the extent to which the initial conditions of the problem match its final conditions. In turn, this specific degree of matching actually corresponds to a decision alternative. The larger the favorable weight space supporting a particular alternative, the greater the matching between the conditions under analysis as framed in the theoretical system. The latter formulates both the alternatives and the decision criteria used for their evaluation. The first application of SMAA-M [18] resorted to the Transaction Cost Theory [19, 20, 21] to illustrate how this technique can support the selection of a supply chain's transaction-cost-minimizing governance forms. This application corresponds to Case 1 of SMAA-M, where the initial and final states are of the same nature [18].

The present work constitutes the second application of SMAA-M and the first one of Case 2, where the initial and final states are not of the same nature. In this regard, the Color Theory utters that each initial state (i.e., learning style) corresponds with a particular final state (i.e., pedagogical strategy). The decision problem is to determine which is the most appropriate pedagogical strategy for the specific learning style of an individual or group of individuals.

In order to model the matching of each alternative, SMAA-M uses a mathematical element called value range (VR), which allows exploring the feasible weight space associated to an alternative's favorability. Each estimation of the feasible weights vector (wherein each weight is associated to a criterion) represents a particular appraisal of the criteria on the part of the DMs, which allows evaluating the alternatives. In turn, a utility function allows identifying alternative dominance in each weight vector, which contributes to the exploration of the matching feasible space of the set of alternatives. In summary, SMAA-M allows computing the convex polytopes of the decision problems, each of which consists in exploring the set of favorable weights of a particular group of VRs, namely those relating the initial states of the system to their corresponding output states, as referenced by the theory.

As to those works addressing educational problems through MCDM [22], have proposed an analytical model to explore key factors affecting the creativity of students through a MCDM approach. In turn [23], have developed a software for appropriate Learning Object (LO) selection by means of AHP. The current literature review failed to find any studies in which decision support systems incorporating theoretical support are employed to select pedagogical strategies, this novelty is introduced in the present work.

## 2 SMAA-M application process

The DM process introduced in the present work comprises four steps:

(1) Identification of the object of study and the theoretical background of the decision problem as framed in the SMAA-M context jointly accepted by the DMs.

This theoretical framework performs as a reference system, one that facilitates the total or partial solution of the problem under study.

(2) Mathematical characterization of the specific application of SMAA-M to the case.

In this step, SMAA-M identifies those Vrs (*R*_*ir*,_) that are related to the alternatives of the decision system as they are introduced by the theoretical paradigm. These *R*_*ir*_ are then associated to the sets and indices of the method, in agreement with the selected reference system.

(3) Obtaining of the input information for SMAA-M

This step allows diagnosing the decision context; obtaining each VR's criterion value matrix, as well as the characteristic range bound matrix; and selecting an adequate procedure to be followed by the DMs in defining SMAA-M's utility functions.

(4) Execution of SMAA-M, resulting in the output data, which is presented to the DMs.

A detailed explanation of SMAA-M is presented in [10].

**Figure 1:**
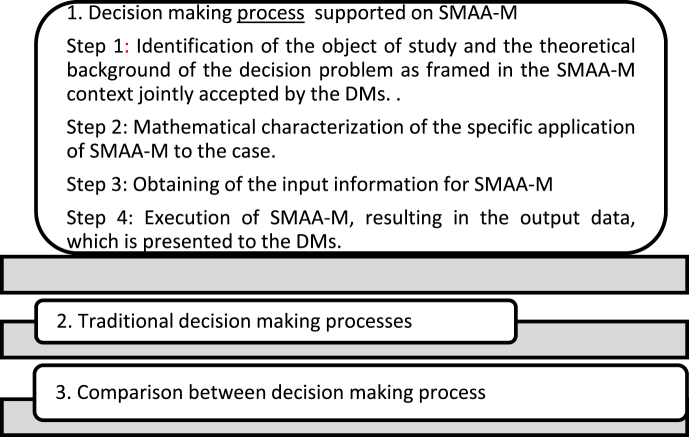
Development of the hypothetical example.

In order to highlight the contribution of the novel DM process, it is compared to the traditional one in section 4, together with the introduction of the hypothetical example, the methodological development of which is shown in Figure 1.

## 3 Hypothetical example

In the lines that follow, the methodological steps of SMAA-M as applied to the studied problem are explained in detail.

### 3.1 Identification of the object of study and the theoretical system that frames the decision problem in the SMAA-M context jointly accepted by the DMs

The theory of colors arises from the concept of Integral Thinking [1, 2], which studies the functioning of the brain. According to this theory, the integral thinking model is defined as the coexistence of four quadrants, as follows:

Left front quadrant (associated to color Blue and labeled “A”): Focused on logical, quantitative and analytical thinking, this quadrant bases its activity on facts and data.
Left rear quadrant (Green – B): This quadrant focuses on organized, sequential, planned and detailed thinking. Rear right quadrant (Red – C): Concerned with emotional, relational and interpersonal thinking. Front right quadrant (Yellow – D): Focused on holistic, intuitive, integral and synthetic thinking.

The concept of Integral thinking has clear correspondence with that of Learning Style [3, 4, 5, 6], which refers to the way a student grasps knowledge. Among those styles we can highlight the following:

• Visual: It is associated to people who have visual verbal or visual spatial preference.
• Auditory: Featured by people who tend to use information in a sequential and ordered way.
• Kinesthetic: Those who prefer this style tend to learn through movement.
• Relational: It uses reflection and interaction with others to learn.
• Logical mathematical: They learn through analysis, reasoning, logic and numbers.

It is fundamentally important for teachers to develop effective pedagogical strategies based on their knowledge about the students and their learning styles. This is so because each learning style requires a particular pedagogical strategy. In this regard, a pedagogical strategy is defined as a set of coherent, ordered and integrated activities developed by the teacher with the aim of accomplishing a particular learning goal for a student or group of students [24]. In providing adequate learning conditions, the following aspects should be considered:

• The way the mind processes and organizes information [8, 25].
• The development of meaningful learning strategies [9, 26].
• The student's active and participating learning role [9, 27].
• The student's features, both individually and as a member of a group [27].
• The supporting role of the teacher in the learning process [6, 28].

In the pursue to develop a comprehensive educational strategy, four major learning groups have been identified through color labels, each of which is linked to set of specific characteristics that allow designing suitably adapted pedagogical strategies [8, 9, 10, 29, 30, 31]. For this purpose, the particular preferences, tendencies, dispositions and behavioral patterns of each student's learning and working mode are taken into account in Table 1, which summarizes the way the Integral Thinking Model is applied to the Learning Process Concept.

**Table 1:** Integral thinking model.

Color	Learning style					
	Visual Verbal	Visual Spatial	Auditory	Kinesthetic	Relational	Logical mathematical
A	2	1	2	0	2	2
B	2	2	1	2	2	0
C	0	2	2	2	2	1
D	1	2	0	2	2	2

Source: Adapted from [1, 2].

In Tables 1 and 2, a score 2 indicates that the pedagogical strategy is likely to be successful for that color, while a score 1 denotes that it should be used with caution, and 0 indicates that its use is not advisable. Table 2 frames the different pedagogical strategies in the Integral Thinking Model.

**Table 2:** Pedagogical strategies.

Criterion (Color) Alternative Pedagogical Strategy	Strategy label	Strategy set label	*A*	*B*	*C*	*D*
Learning through projects	1	*5*	2	2	2	2
Learning through experience – workshops	2	*3*	1	2	2	1
Magisterial class – questions	3	*3*	2	1	1	2
Guided laboratory practice	4	*3*	1	2	2	1
Learning through problems	5	*2*	2	0	1	2
Learning through experience – role games	6	*2*	1	0	2	2
Learning through experience – simulation	7	*2*	2	1	0	2
Case study	8	*4*	2	2	2	1
Magisterial class – Audiovisual	9	*2*	1	2	1	1
Lectures	10	*2*	2	1	1	1
Readings	11	*2*	2	1	1	1
Tutorials	12	*1*	1	2	1	0
Presentations	13	*2*	2	1	1	1
Autonomous activities	14	*1*	1	1	1	1
Learning through cooperation	15	*1*	0	1	2	1
Magisterial class – Presentation	16	*1*	1	2	1	0
Tasks	17	*1*	1	1	1	1

Source: Adapted from [1, 2, 8, 9, 10].

Table 2 presents the pedagogical strategies and their level of adaptability to each thinking style (0: low, 1: medium, 2: high) [see four last columns and details in the third column (pedagogical strategy sets)]: *1* - (12, 14–17); *2* - (5–7, 9–11, 13); *3* - (2–4); *4* - (8); *5* – (1). These group the adequate strategies for each color (learning style), as synthesized by the theoretical references [1, 2, 3, 4].

Table 3 presents the pedagogical strategies recommended for each combination of thinking styles. The columns illustrate the dominant color, whereas the lines show the secondary color [1, 2, 3, 4]. According to [32], over 60% of people are skillful at performing in the areas identified by two colors.

**Table 3:** Recommended pedagogical strategies according to student learning styles.

A	B	C	D	
	Magisterial class – Presentation	Learning through cooperation	Learning through problems	*A*
Magisterial class – questions		Case study	Learning through experience – simulation	*B*
Learning through projects	Guided laboratory practice		Learning through experience – role games	*C*
Learning through problems	Tutorials	Learning through experience – role games		*D*
Learning through cooperation	Learning through experience – role games	Learning through experience – simulation	Tutorials	Not recommended
Learning through experience – role games	Learning through problems	Tutorials	Magisterial class – Presentation	Not recommended

Source: Adapted from [1, 2, 3, 4, 8, 9, 10].

As to illustrate the application of SMAA-M to this educational issue, a hypothetical example was proposed, which simulated the thinking style of a student. Nevertheless, the same process can be applied to a group of students. Thinking styles were deduced through instruments especially developed by sources such as [33].

The analytical model presented in Tables 2 and 3 (above) was used as decision reference for the analysis, whose result allowed the student to determine the set of pedagogical strategies that would suit them better.

In the present case, the weights of each criterion were estimated by means of a [0, 1] uniform distribution. The sum of these weights satisfies the convexity constraint of the polytope, inasmuch as it is not larger than 1.

### 3.2 Mathematical characterization of the specific application of SMAA-M to the case

In the situation dealt with in the present work, the input states of the system (which correspond to a particular student) are different in nature from its output states (i.e., the ideal pedagogical strategies for the student). The current example focuses on just one student (input state *i* = *1*), which is enough to illustrate the application of SMAA-M, since the technique is robust enough to handle a large number of input states (student cases). The number of criteria (*j*) goes from *1* to *4*, since it is associated to the four major learning styles of the educational model, which are identified by the color features. In turn, the number of output states (*r*), which correspond to the set of pedagogical strategies proposed by the educational model, actually range from *1* to *5*. In this way, an illustrative set *R*_*ir*_ is made up of 5 VRs, (*1*, *1*),…, (*1*, *5*), each of which is evaluated by the set of criteria of the learning styles. In this particular context, each VR combines both the student's learning style and the pedagogical strategies that fit him/her.

### 3.3 Obtaining SMAA-M's input information

Finally, in both cases (opinion values and range bounds), a standardization process was carried out by mapping the values resulting from the sum on a [0,1] uniform distribution function. The VRs.

The analysis performed through SMAA-M sought to determine the student's acceptability of the different learning styles (colors). The following list presents the student's characteristic opinion values for each of the criteria. As it can be seen in Table 4, the student has a relatively strong performance with regard to the four colors, especially criteria B, C and D, as it can be deduced from the reference presented in Table 2.

**Table 4:** Standardized and non-standardized deterministic criterion values for each alternative.

v_ij_					
	*i*	*j*			
		*A*	*B*	*C*	*D*
***Criterion***	***1***	1.28	1.78	1.68	1.74
***Standardized criterion***		0.64	0.89	0.84	0.87

The associated range bounds as defined for the present study are shown in Table 5. These were determined through the standardized average of the criterion values associated to the colors that feature the theoretical model (see Table 2). In this particular case, each *ε*→ 0, which corresponds to the limit between the bounds of each VR.

**Table 5:** *VR* characteristic bounds.

(*y*_*i*_^*r*^*, x*_*i*_^*r*^)					
i	r				
	1	2	3	4	5
***1***	[0, 0.5)	[0.5, 0.63)	[0.63, 0.75)	[0.75, 0.88)	[0.88, 1]

(4). *Execution of SMAA-M, resulting in the output data presented to the DMs.*

The acceptability indexes estimated for each range are presented in Table 6.

**Table 6:** Acceptability indexes.

a_i_^r^					
*i*	*r*				
	*1*	*2*	*3*	*4*	*5*
***1***	0	0	0.1167	0.8806	0.0027

From the table it can be mainly concluded that the student, should preferably use the pedagogical strategies related to output state *4*, which contains pedagogical strategy 8 (Case study). As secondary alternatives, the student could resort to the pedagogical strategy set related to output state *3.* This specific set includes pedagogical strategies 2 (Learning through experience – workshops), 3 (Magisterial class - questions) and 4 (Guided laboratory practice). This is so because the set of feasible weights is almost completely filled by supporting output states 4 and 3, the former one being remarkably larger than the latter. Alternative 5 is only marginally supported, while alternatives 1 and 2 are not supported.

The average weight vectors presented in Table 7 show that the weights supporting output state *4* have similar values due to the fact that the criteria associated to the decision problem are equivalently important, except for criterion *1*, which shows a lower value. The other decision ranges show biased features, where criterion *A* is dominant for output state *3*, and criterion *B* is dominant for output state *5*.

**Table 7:** Central weights.

wc_i_^r^	j = A	j = B	j = C	j = D
*Wc*_*1*_^*3*^	0.631	0.093	0.157	0.118
*Wc*_*1*_^*4*^	0.191	0.267	0.270	0.271
*Wc*_*1*_^*5*^	0.011	0.826	0.046	0.117

To conclude the analysis, the favorable weight bounds are presented in Table 8. Just as in Table 5, there are no bound indexes for ranges *1* and *2*, because the favorable weight sets are empty. As it can be expected, the different indicators are consistent with each other and lead to the same conceptual result.

**Table 8:** Weight bounds.

Max (Min)_j_ {*w*_*ij*_^*r*^}												
w_i_^j^	w_1_^3^				w_1_^4^				w_1_^5^			
j	A	B	C	D	A	B	C	D	A	B	C	D
Max _*j*=*A*_	1	0	0	0	0.56	0.44	0	0	0.04	0.96	0	0
Min _*j=A*_	0.45	0	0.55	0	0	0	0	1	0	0.5	0	0.5
Max _*j=B*_	0.56	0.44	0	0	0.04	0.96	0	0	0	1	0	0
Min _*j=B*_	0	0.52	0	0.48	0	0	0	1	0	0.5	0	0.5
Max _*j=C*_	0.45	0	0.55	0	0	0	1	0	0.2	0	0.8	0
Min _*j=C*_	0.52	0	0	0.48	0	0	0	1	0	0.5	0	0.5
Max _*j=D*_	0.52	0	0	0.48	0	0	0	1	0	0.5	0	0.5
Min _*j=D*_	0.45	0	0.55	0	0	0	1	0	0	0.8	0.2	0

### 3.4 Contrast analysis and determination of the value added by the proposal

In order to assess the efficacy of the current SMAA-M supported DM process, a reference analysis was carried out, framed in the theoretical paradigm shown in Table 3. From said analysis, it can be inferred that the most favored pedagogical strategies are: Learning through experience - simulation, Learning through experience - role games, Case study, Guided laboratory practice, and Tutorials. These were obtained by the combination of the main colors for the case (*B*, *C* and *D*), since they showed the highest and most similar values when compared to *A*.

The comparison between the DM process supported by the theoretical reference in Table 3 and the one supported by SMAA-M shows several coincidences in terms of the main strategies. This comparison is complemented by the more complete theoretical reference shown in Table 2. However, the SMAA-M supported process allows prioritizing the pedagogical strategies, out of which the case study was the most favored one. This prioritization cannot be provided by the traditional process, as illustrated in Table 3. Additionally, SMAA-M allowed inferring that the secondary pedagogical strategies determined in Table 3 are not recommended for the object of study. In summary, although there are important coincidences, SMAA-M allows the development of a more adjusted analysis of the pedagogical strategies, which represents a significant added value that justifies its use for the solution of the problem.

## 4 Conclusions

The most important contributions made by the current work are: (i) A DM Process to identify appropriate pedagogical strategies, framed in the theoretical paradigm provided by the Theory of Colors and the Learning Styles concept; and (ii) introducing the first application of SMAA-M's Case 2.

SMAA-M showed important advantages over the traditional analysis and DM process.

New practical applications of the technique can be explored on the grounds provided by the present work. The current application of the technique to the theoretical approaches in question may have fruitful consequences that are still being explored and cannot be fully appreciated at the moment. In this regard, the results and the technique employed in this work can be easily compared and complemented with other studies using similar theoretical frameworks and related techniques. In this sense, SMAA-M could undertake analyses that integrate other theoretical paradigms to define pedagogical strategies. As such the current method could provide a reference for other technical perspectives that deal with the problems discussed here, such as the multivariate statistics dossier.

## Declarations

### Author contribution statement

Rafael Guillermo García-Cáceres: Performed the experiments; Analyzed and interpreted the data; Contributed reagents, materials, analysis tools or data; Wrote the paper.

John Wilmer Escobar-Velásquez, Arnoldo Emilio Delgado-Tobón: Conceived and designed the experiments; Contributed reagents, materials, analysis tools or data.

### Funding statement

This research did not receive any specific grant from funding agencies in the public, commercial, or not-for-profit sectors.

### Data availability statement

Data included in article/supplementary material/referenced in article.

### Declaration of interests statement

The authors declare no conflict of interest.

### Additional information

No additional information is available for this paper.
